# Fruiting Branch K^+^ Level Affects Cotton Fiber Elongation Through Osmoregulation

**DOI:** 10.3389/fpls.2016.00013

**Published:** 2016-01-22

**Authors:** Jiashuo Yang, Wei Hu, Wenqing Zhao, Binglin Chen, Youhua Wang, Zhiguo Zhou, Yali Meng

**Affiliations:** Key Laboratory of Crop Physiology and Ecology, Department of Agronomy, College of Agriculture, Nanjing Agricultural UniversityNanjing, China

**Keywords:** cell turgor, cotton, fiber elongation, K deficiency, [K^+^]_FB_, [K^+^]_fiber_, *V*_max_

## Abstract

Potassium (K) deficiency in cotton plants results in reduced fiber length. As one of the primary osmotica, K^+^ contributes to an increase in cell turgor pressure during fiber elongation. Therefore, it is hypothesized that fiber length is affected by K deficiency through an osmotic pathway, so in 2012 and 2013, an experiment was conducted to test this hypothesis by imposing three potassium supply regimes (0, 125, 250 kg K ha^-1^) on a low-K-sensitive cultivar, *Siza 3*, and a low-K-tolerant cultivar, *Simian 3*. We found that fibers were longer in the later season bolls than in the earlier ones in cotton plants grown under normal growth conditions, but later season bolls showed a greater sensitivity to low-K stress, especially the low-K sensitive genotype. We also found that the maximum velocity of fibre elongation (*V*_max_) is the parameter that best reflects the change in fiber elongation under K deficiency. This parameter mostly depends on cell turgor, so the content of the osmotically active solutes was analyzed accordingly. Statistical analysis showed that K^+^ was the major osmotic factor affecting fiber length, and malate was likely facilitating K^+^ accumulation into fibers, which enabled the low-K-tolerant genotype to cope with low-K stress. Moreover, the low-K-tolerant genotype tended to have greater K^+^ absorptive capacities in the upper fruiting branches. Based on our findings, we suggest a fertilization scheme for *Gossypium hirsutum* that adds extra potash fertilizer or distributes it during the development of late season bolls to mitigate K deficiency in the second half of the growth season and to enhance fiber length in late season bolls.

## Introduction

Potassium (K) is a critical macronutrient for cotton production as it is vital to enzyme activation, electrochemical gradient maintenance, cell turgor generation, and osmotic regulation (Marschner, 1995; Amtmann and Blatt, 2008; Zhang et al., 2009; Römheld and Kirkby, 2010). Cotton (*Gossypium hirsutum* L.) is more sensitive to K deficiency compared to other crops (Cope, 1981; Cassman et al., 1990) because its developing bolls act as strong K sinks (Boquet and Moser, 2003). As a consequence of the constant decrease in soil available K caused by improved yields and insufficient K application, an increasing number of K deficiency events have been affecting cotton production worldwide (Oosterhuis et al., 1994). This issue has become a limiting factor for cotton production in China (Zhang et al., 2007).

Field tests have shown that soil K deficiency not only reduces K concentrations in cotton leaves (Reddy and Zhao, 2005), bolls (Wright, 1999), and fibers (Cassman et al., 1990), but it also affects a series of fiber quality parameters (Cassman et al., 1990; Pettigrew et al., 1996), especially fiber length (Bauer et al., 1998). *In vitro* research has demonstrated a specific K requirement for cotton fiber elongation (Dhindsa et al., 1975), and because K^+^ is one of the primary osmotically active solutes in fiber cells that powers elongation (Dhindsa et al., 1975; Ruan, 2005), fiber length is very likely to be affected by K deficiency through an osmoregulatory pathway. However, no direct evidence currently exists to prove this hypothesis. Additionally, Cassman et al. (1990) indicated that fiber length was positively related to fiber K^+^ concentration at maturity, whereas the K^+^ in fiber also plays a major role before 25 days postanthesis (DPA; Dhindsa et al., 1975). Therefore, it might be useful to uncover the quantitative relationship between fiber length and fiber K^+^ level during the rapid elongation stage.

Although it usually takes 40–60 days for cotton fiber to reach maturity, its final length is attained within the first 3 weeks (Ruan, 2005). Recent research has shown that adjacent elongating fibers are joined together by the adhesion of cotton fibre middle lamella (CFML) into tissue-like bundles (Avci et al., 2013), while the fiber cell elongation process follows a single-cell expansion mode (Ruan et al., 2001; Ruan, 2005; Qin and Zhu, 2011). Two inner factors have been found that can regulate fiber elongation (Braden and Smith, 2004; Zhao et al., 2012): (1) the force of cell turgor against cell walls to achieve cell expansion (Ruan et al., 2001; Fricke and Peters, 2002) and (2) the duration of cell turgor (Ruan et al., 2001; Ruan, 2005). Cell turgor is achieved through the influx of water, which is driven by osmotically active solutes (Cosgrove, 1997). Soluble sugars, K^+^ and malate are the three main osmotically active solutes in elongating fibers, which together account for approximately 80% of the total osmotic potential (Dhindsa et al., 1975; Ruan et al., 1997). To achieve elongation, highly expressed endo-1,4-glucanase, expansin proteins, etc., contribute to unlocking the network of cell wall polysaccharides, which permits turgor to drive fiber cell elongation (Shimizu et al., 1997; Ruan et al., 2001; Harmer et al., 2002). However, the down-regulation of these proteins during the elongation phase would hinder fiber cell lengthening (Frederic et al., 1998; Cosgrove, 2000). Secondary cell wall formation would also regulate fiber cell elongation, which usually starts at 16–20 DPA (days postanthesis) and, compared to the early stage, increases the cellulose synthesis rate by over 100-fold, which ultimately makes the cell wall too rigid to elongate (Ruan, 2007; Wang and Ruan, 2010). Additionally, plasmodesmata (PD) have been found to be gateways that control the generation of fiber cell turgor (Ruan et al., 2001). Although all of the factors mentioned above can regulate the fiber elongation process, only the accumulation of osmotic solutes can drive this process (Delmer, 1999; Martin et al., 2001; Ruan, 2005). Therefore, it is logical to study the mechanism by which K^+^ level affects fiber length.

Cotton bolls grown on the upper fruiting branches (late season bolls) are more prone to symptoms of K deficiency (Read et al., 2006). Previous research has shown that soil K deficiency reduced fiber length by 5.0–7.6% in late season bolls, but this reduction was only 0–1.8% in early season bolls (Read et al., 2006). More information is needed to understand fiber length on a whole plant level. In consideration of the issues mentioned above and some other key points, a 2-year test has been undertaken on a couple of severely K-deficient fields. This study aimed to determine: **(a)** the characteristics of the changes that occur during fiber elongation under K deficiency; **(b)** the effect of plant K^+^ status on cotton fiber elongation and the relationship between [K^+^] in tissues and fiber elongation properties; **(c)** the reason why late season bolls are sensitive to K deficiency; **(d)** the mechanism underlying genotypic differences in the capacity for low-K tolerance.

## Materials and Methods

### Cultivar and Site Description, Experimental Design and Crop Management

Two cotton (*G. hirsutum* L.) cultivars, *Simian 3* and *Siza 3*, have been identified as low-K-tolerant and low-K-sensitive genotypes, respectively (Yang et al., 2014, doi: 1002-7807(2014)04-0301-09, Chinese version with English abstract, see Supplementary Figure S1). Cotton was planted in a purpose-built test field that had been potassium (K) depleted for two crop seasons, which is located at the Pailou site (yellow–brown loam) of Nanjing Agricultural University, Jiangsu Province, China. Cotton was sown on 25th Apr and transplanted on 22 May in both 2012 and 2013. The soil soluble K^+^ concentrations (0–40 cm) before planting in 2012 and 2013 were 92 and 86 mg kg^-1^, respectively. To avoid the effects of the fertilizer residues from the first year on the experiment in the second year, we separated the test field into two parts for each planting.

Three K fertilizer application levels of 0, 125, 250 kg K ha^-1^ per year were arranged in a randomized complete-block design with three replicates. No K applied combined with K-deficient soil created a severely K-deficient condition while the 125 and 250 kg K ha^-1^ treatments induced mild K deficiency and K sufficiency, respectively (Gormus, 2002; Pettigrew, 2008). Individual treatment plots were 6.6 m × 13 m size with 16 rows each, and K fertilizer (potassium sulfate, K content = 42%) was accurately hole-applied at the beginning of flowering season in each year. Nitrogen fertilization (240 kg N ha^-1^ per year, urea), phosphate fertilization (52 kg P ha^-1^ per year, calcium superphosphate), irrigation, pest control, etc., followed recommended practices. There were no indications that N supply, water stress or pest pressure negatively influenced cotton growth during the 2 years of the experiment.

### Sampling Procedure and Measurement of Fiber Length

The first fruit-bearing sympodial branch was defined as the first fruiting branch (FB_1_). Generally, based on their growth habits and the climate in Nanjing, China, these two cultivars would have 14–16 fruiting branches before artificial topping. Therefore, the 3rd, 8th, and 13th fruiting branches of the cotton plant were tagged as representatives of the lower, middle, and upper fruiting branches, respectively. Each FB sample was cut from three replicated plants to remove the bolls, leaves, flowers, and buds before oven drying at 105°C for 0.5 h and then at 70°C to a constant weight before grinding into powder for use. All soil samples (0–40 cm) were collected from nine separate locations from each plot and mixed together and naturally air-dried to a constant weight followed by grinding into powder for use. Both the fruiting branches and soil samples were collected on 25 July, 5 August, 15 August, 25 August, and 5 September, which corresponded to 63, 74, 84, 94, and 105 days after transplantation (22 May) in both years. Cotton boll age was determined by tagging the fully opened white flower (onset of anthesis, 0 DPA).

For the measurement of immature fiber length at 5, 10, 15, 20, 24, 31, 38, and 45 DPA, bolls grown on the 3rd, 8th, and 13th fruiting branches were collected (plus an additional 17-DPA sample for the eight FB). Cotton bolls were picked and stored on ice for no more than 1 h before dissection, and bolls younger than 30 DPA were boiled with their locules in 0.1% HCl to separate the seeds and relax the fibers (Schubert et al., 1973). Thereafter, fiber length was measured by the water stream method (Thaker et al., 1989). Each seed was carefully placed on the convex surface of a watch-glass, and the fibers on seed were then streamed out with a jet of water. Fiber length was measured with a digital vernier caliper, which could be accurate to 0.01 mm, under a dissecting microscope. Measurement began from the attachment point on the epidermis of the ovule to the edge where most fibers terminate. Ten seeds were randomly selected from three bolls as replicates for the measurements.

A photoelectric stapler (Y-146 Photoelectric Stapler, Taicang Electron Apparatus Co., Ltd., Taicang, Jiangsu, China) was used to measure the length of fibers older than 30 DPA. Prior to measurement, these fiber samples were preprocessed by an oven dryer at 60°C for 0.5 h, then at 40°C for 2 days, and then placed into a standard testing room with constant temperature (20 ± 2°C) and humidity (65 ± 2%) for 48 h. Fiber samples from 30 DPA were measured by both methods to correct for differences between the two systems.

### K^+^ Concentration Analyses

Fiber samples younger than 30 DPA were divided into those used for the fiber length measurements and the remainder, which were oven-dried through the same fiber-drying processes described above. Both the fiber and FB samples were digested by H_2_SO_4_-H_2_O_2_ (Samal et al., 2010), and soil soluble K^+^ was extracted by placing 2 g prepared soil into 20 ml 1 M NH_4_OAc at pH 7.0 and shaking the flask at 200 rpm for 5 min followed by a filtration process (Warncke and Brown, 1998). The K^+^ concentrations in the sample solution were measured by an atomic absorption spectrophotometer (TAS-986 Atomic Absorption Spectrophotometer, Beijing PERSEE Co., Ltd., Beijing, China), and [K^+^]_fiber_, [K^+^]_FB_ and [K^+^]_soil_ were all calculated based on the dry weight.

### Malate and Soluble Sugar Concentration Analyses

The extraction method combined with the measurement of fiber malate content was based on Famiani et al. (2000), and the soluble sugar determination method was based on Jones et al. (1977) and Famiani et al. (2000). In summary, 50 mg of a dry fiber sample were frozen by liquid nitrogen and ground into powder and then placed in 1.5 ml of 80% ethanol containing 100 mM Hepes-KOH (pH 7.1) and 20 mM MgCl_2_ for 1 h at 80°C for extraction. The extract was centrifuged at 12 000 *g* for 5 min after cooling. The supernatant was recovered, and 150 μl of charcoal suspension (100 mg ml^-1^) was added with stirring, after which another centrifugation with the same settings was undertaken. The last supernatant was the extract.

Glucose, fructose, and sucrose contents were measured using the enzyme-coupled method (Jones et al., 1977; Famiani et al., 2000). First, 200 μl of extract was added into the 1 ml assay buffer containing 100 mM Hepes-KOH (pH 7.0), 5 mM MgCl_2_, 0.5 mM DTT, 0.02% (w/v) BSA, 100 mM ATP, and 40 mM NAD^+^. Then, glucose was measured by initiating the reaction by adding 3 U (in 100 μl) of hexokinase (from *Saccharomyces cerevisiae*, Sigma Co.) and 1 U (in 100 μl) of glucose-6-phosphate dehydrogenase (from *Leuconostoc mesenteroides*, Sigma Co.) into the assay mixture. After 30 min at 30°C, the absorption was measured at 340 nm, and the standard curve was made by replacing the sample extraction with a series of glucose solutions (prepared in extraction buffer). Fructose and sucrose were sequentially analyzed after glucose following the addition of 1 U (in 100 μl) of phosphoglucose isomerase (from *S. cerevisiae*, Sigma Co.) and 100 U (200 μl) of invertase (from *S. cerevisiae*, Sigma Co.), respectively, to the assay mixture. The same detection method was used for fructose and sucrose determination as for glucose. The measurement principle was based on Jones et al. (1977).

Malate (Famiani et al., 2000) was measured by adding 200 μl of extract into 1 ml of assay buffer containing 50 mM 2-amino-2-methylpropanol, 40 mM glutamate (pH 9.9), and 1 mM NAD^+^. The reaction was initiated by adding 10 U (in 100 μl) of glutamate-oxaloacetate transaminase (GOT, from pig heart, Sigma Co.) and 0.7 U (in 100 μl) of L-malate dehydrogenase (L-MDH, from pig heart, Sigma Co.) into the assay mixture. After 1 h at 20–25°C, the absorption was measured at 340 nm, and the standard curve was built by replacing the sample extract with a series of malate solutions (prepared in extraction buffer).

### Statistical Analyses

ANOVA was performed using SPSS 20 (IBM). **Figures 1–9** were drawn with Origin 9.0 (OriginLab), and **Figure 10** was drawn with PowerPoint (Microsoft).

**FIGURE 1:**
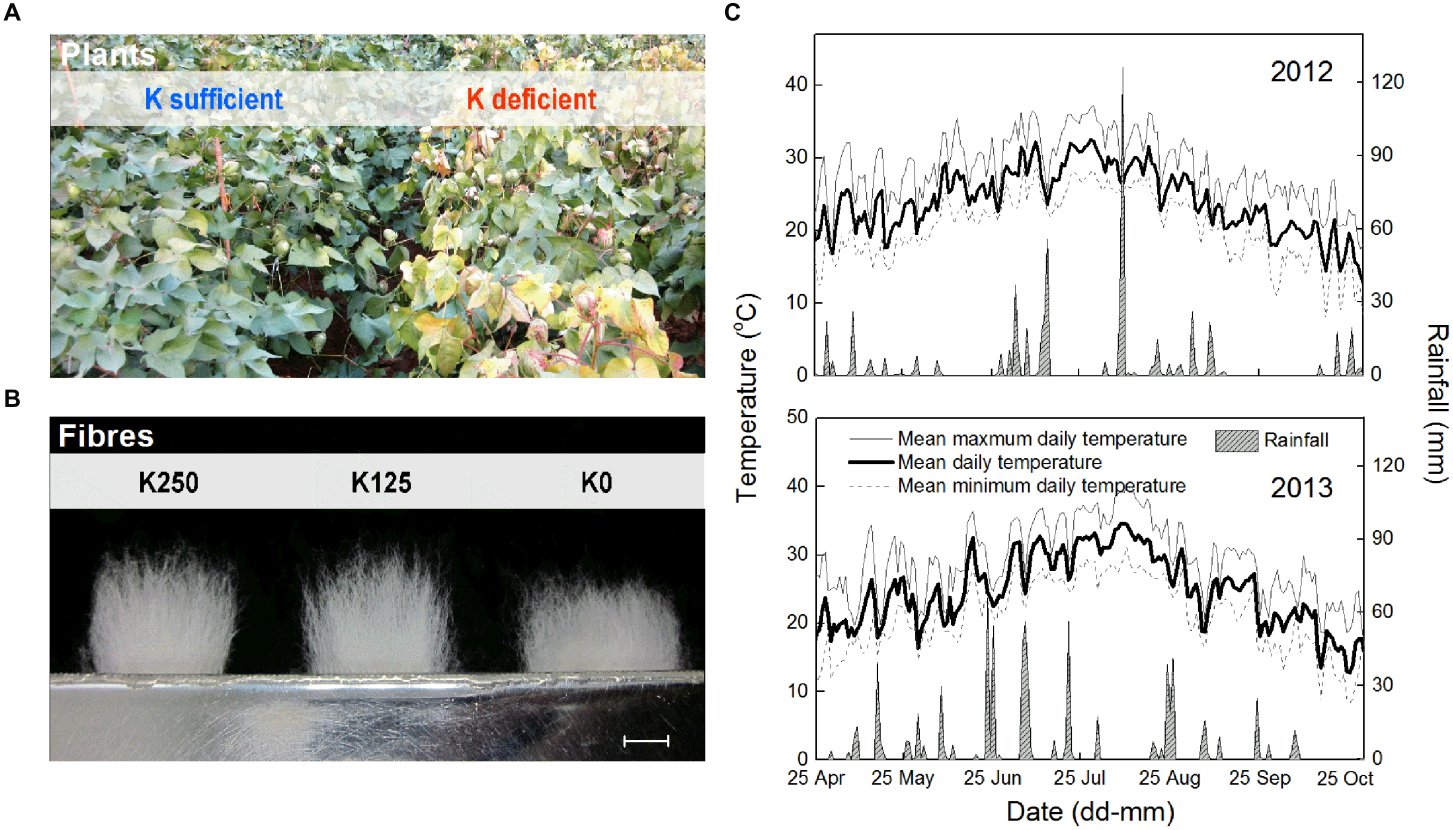
**K deficiency symptoms in cotton plant (A), cotton fibers (B), and growing season temperatures and rainfall (C).** Fibers shown in **(B)** were ginned from the 8th fruiting branch (FB_8_) of *cv. Siza 3* and were tidied by a pair of special combs. Bar = 10 mm. K250, K125, and K0 represent 250, 125, and 0 kg K ha^-1^, respectively.

**FIGURE 2:**
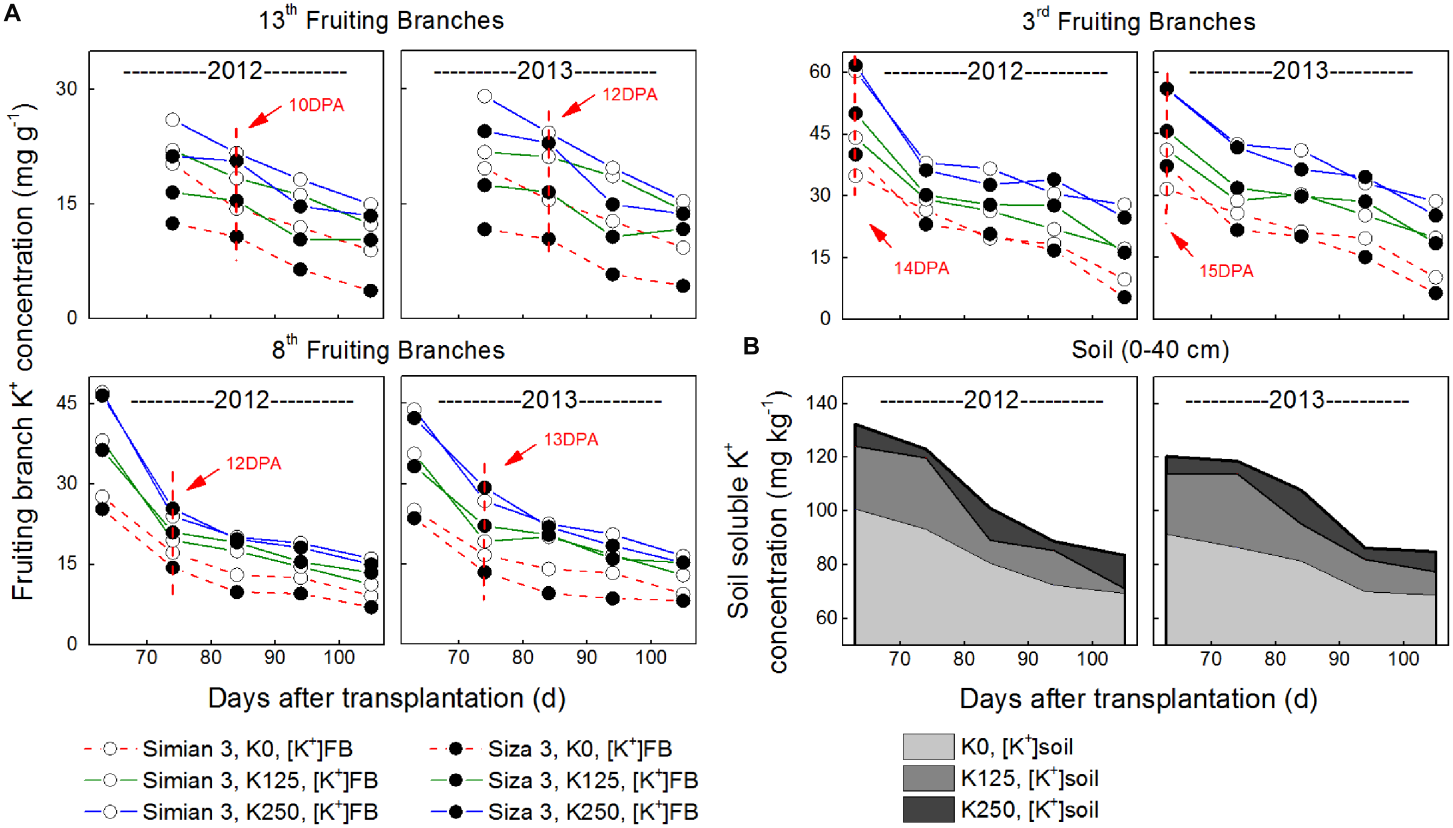
**Changes in FB K^+^ concentration ([K^+^]_FB_, A) and soil soluble K^+^ concentration ([K^+^]_soil_, B) during cotton growth.** Each data point in **Figure 2** was determined from a sample mixture of three replicate plants **(A)** or nine points in one plot **(B)**.

**FIGURE 3:**
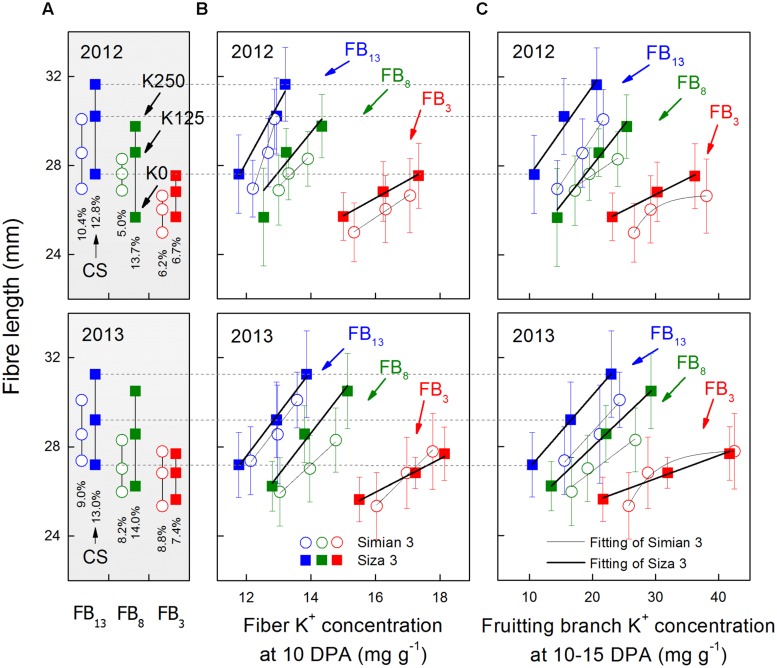
**Changes in final fiber length (A) and its relationship with fiber K^+^ concentration ([K^+^]_fiber_) at 10 DPA (B) and FB K^+^ concentration ([K^+^]_FB_) at 10–15 DPA (C).** CS (coefficients of stress) was calculated by the formula [*Len*_fibre(K250)_*-Len*_fibre(K0)_)*/Le*n_fibre(K250)_ × 100%, in which *“Len*_fibre_*”* represents fiber length. The [K^+^]_fiber_ data presented above were collected at 10 DPA as [K^+^]_fiber_ reached its peak value at approximately 10 DPA (see **Figure 4**). The [K^+^]_FB_ data were collected on 25 July for FB_3_, on 5 August for FB_8_ and on 15 August for FB_13_, which correspond to the 10–15 DPA for the bolls grown on each FB (see arrows in **Figure 2A**). Bars represent ±SE (10 replicates).

**FIGURE 4:**
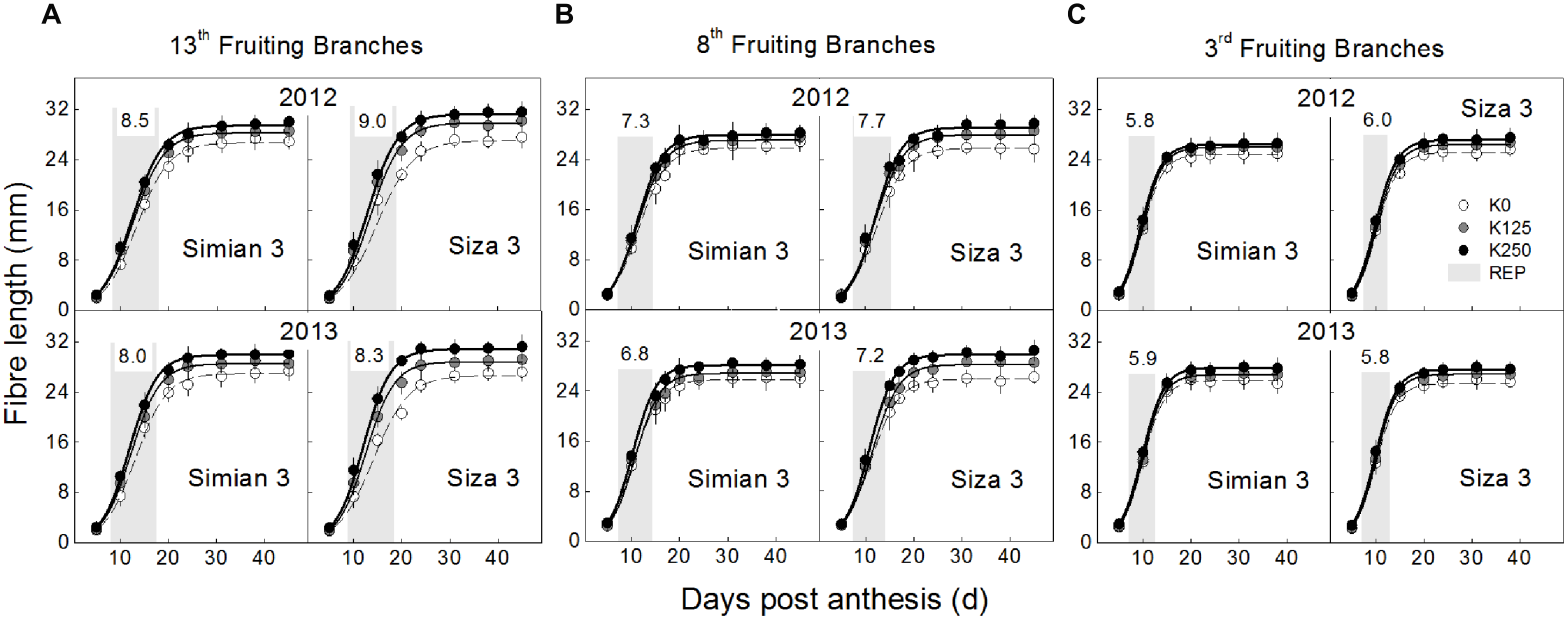
**Dynamic changes in fiber length from 5 DPA to boll opening, which grown on the 13th (A), 8th (B) and 13th (C) fruiting branches.** Each data point represents the mean value from 10 seeds (ovules) with three measurements per seed. Bars represent ±SE (30 replicates).

**FIGURE 5:**
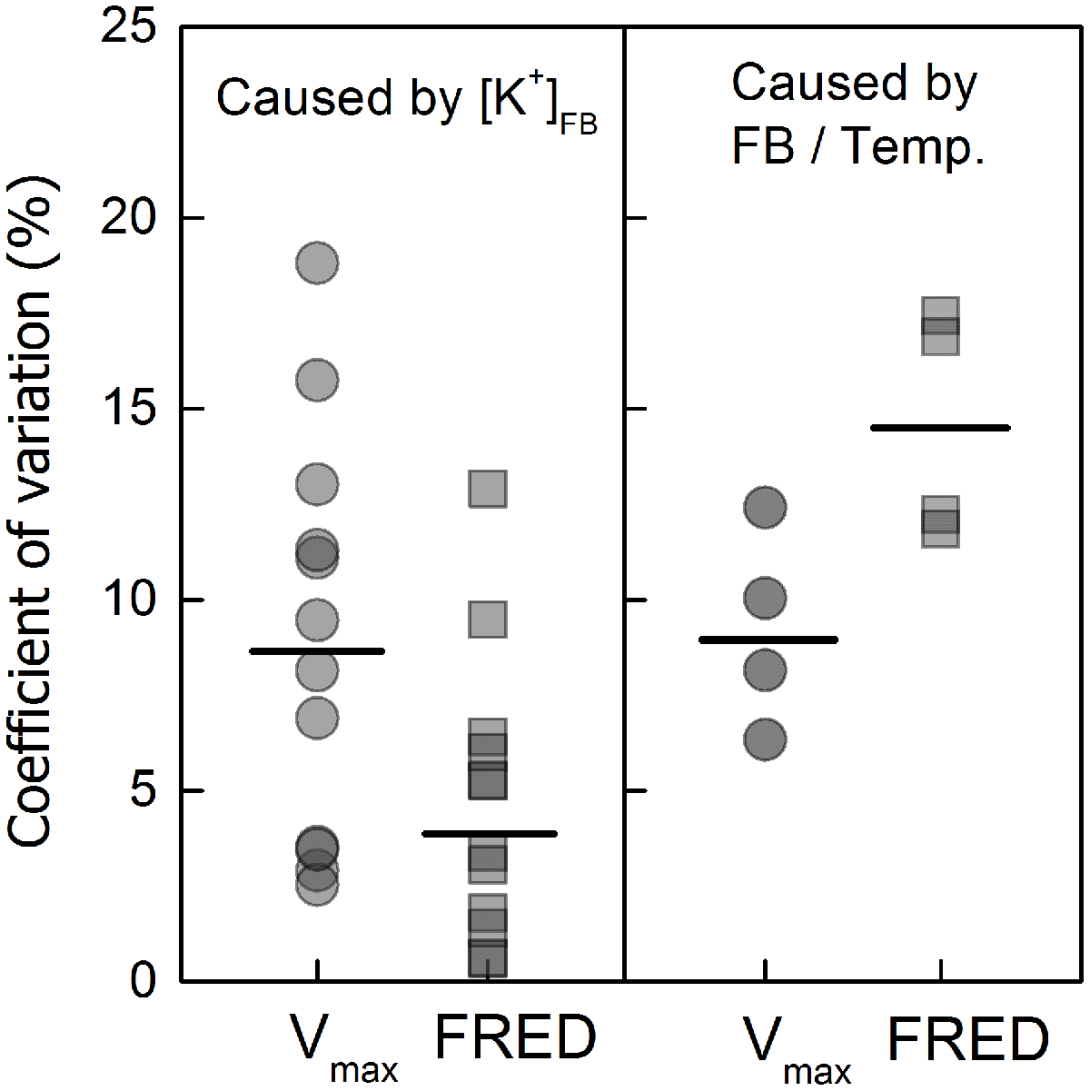
**Coefficients of variation (CV) of “the maximum velocity of fibre elongation” (*V*_max_) and “the fibre rapid elongation duration” (FRED) caused by the varying FB K^+^ concentrations ([K^+^]_FB_) or “FB/boll period temperature” (FB/temp.) ratios.** The CV was calculated by the formula *SD/Mean × 100%*, and the “FB/temp.” factor represents the variation in FB that appeared with variation in boll period temperature. The mean CV value (short black bar) caused by [K^+^]_FB_ (the left side) was calculated by combining the cultivars, test years and fruiting branches; the value caused by “FB/Temp.” (the right side) was calculated by combining the cultivars and test years and only accounted for K-sufficient cotton (K250).

**FIGURE 6:**
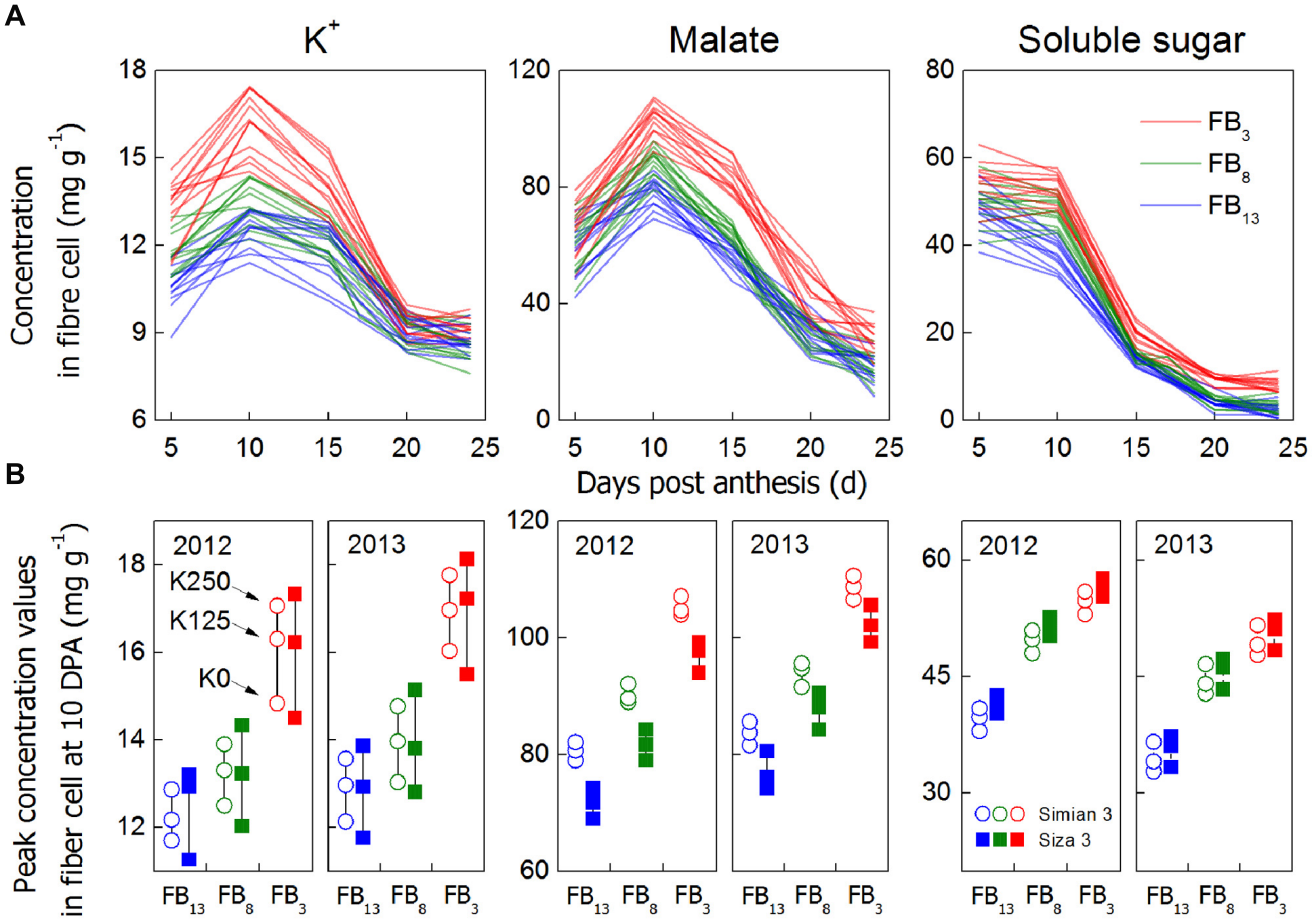
**Concentrations of main osmotically active solutes in cotton fiber (mg g^-1^ DW) from 5 to 24 DPA (A) and the results of the peak value analysis (10 DPA, B).** Each data point is an average of three biological replicates. For **(A)**, we combined all of the data from the three K treatments, three branches, two cultivars and two test years into one color with the aim of detecting their peak values. Peak values **(B)** were observed from 10 DPA because both the peak values and *V*_max_ (**Figure 4**) consistently emerged at approximately 10 DPA. The reason for choosing soluble sugar concentration at 10 DPA as a peak value instead of 5 DPA was that the fibers did not elongate rapidly at 5 DPA. Even if there was a higher value at 5 DPA, it had no significant meaning for the elongation process. Additionally, the relatively higher value at 5 DPA might be caused by the difficulty stripping fiber at 5 DPA, so that some fiber maybe have ovule tissue residue. The three points aligned in a row **(B)** represent fibers on the same FB, from the same cultivar, in the same year but subject to different K treatments.

**FIGURE 7:**
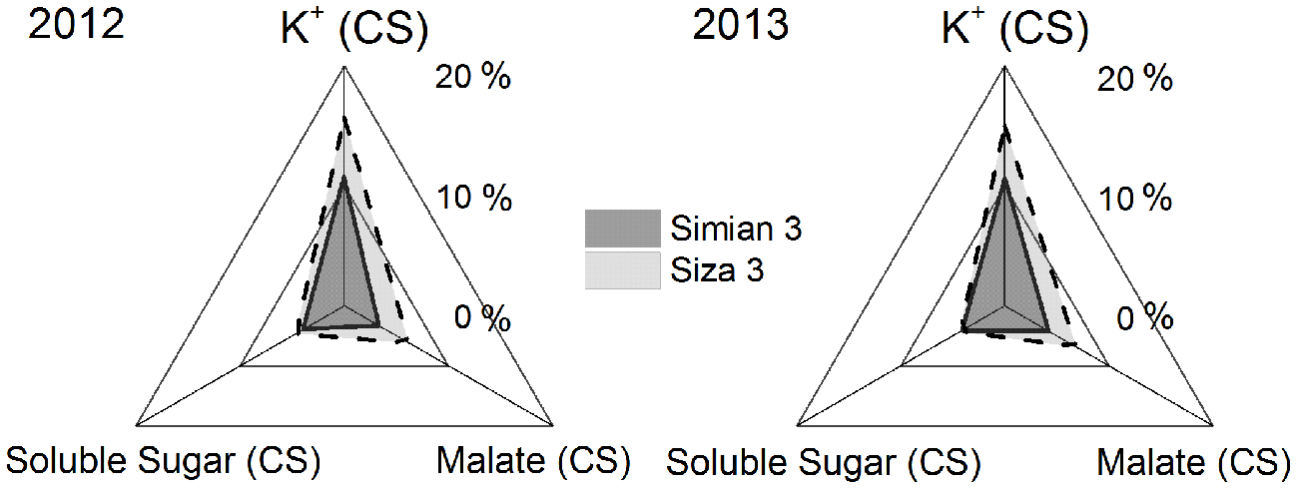
**Coefficients of stress (CSs) of the concentrations of the main osmotically active solutes in fibers at 10 DPA when suffering from K deficiency.** CS was calculated by the formula [*C*_i(K250)_ *- C*_i(K0)_)*/C_i_*_(K250)_ *× 100%*, in which *“C”* represents the concentration at 10 DPA, and *“_*i*_”* could be fiber K^+^, malate or soluble sugar. CS was calculated regardless of FB, which means it was the mean value of the data from three fruiting branches.

**FIGURE 8:**
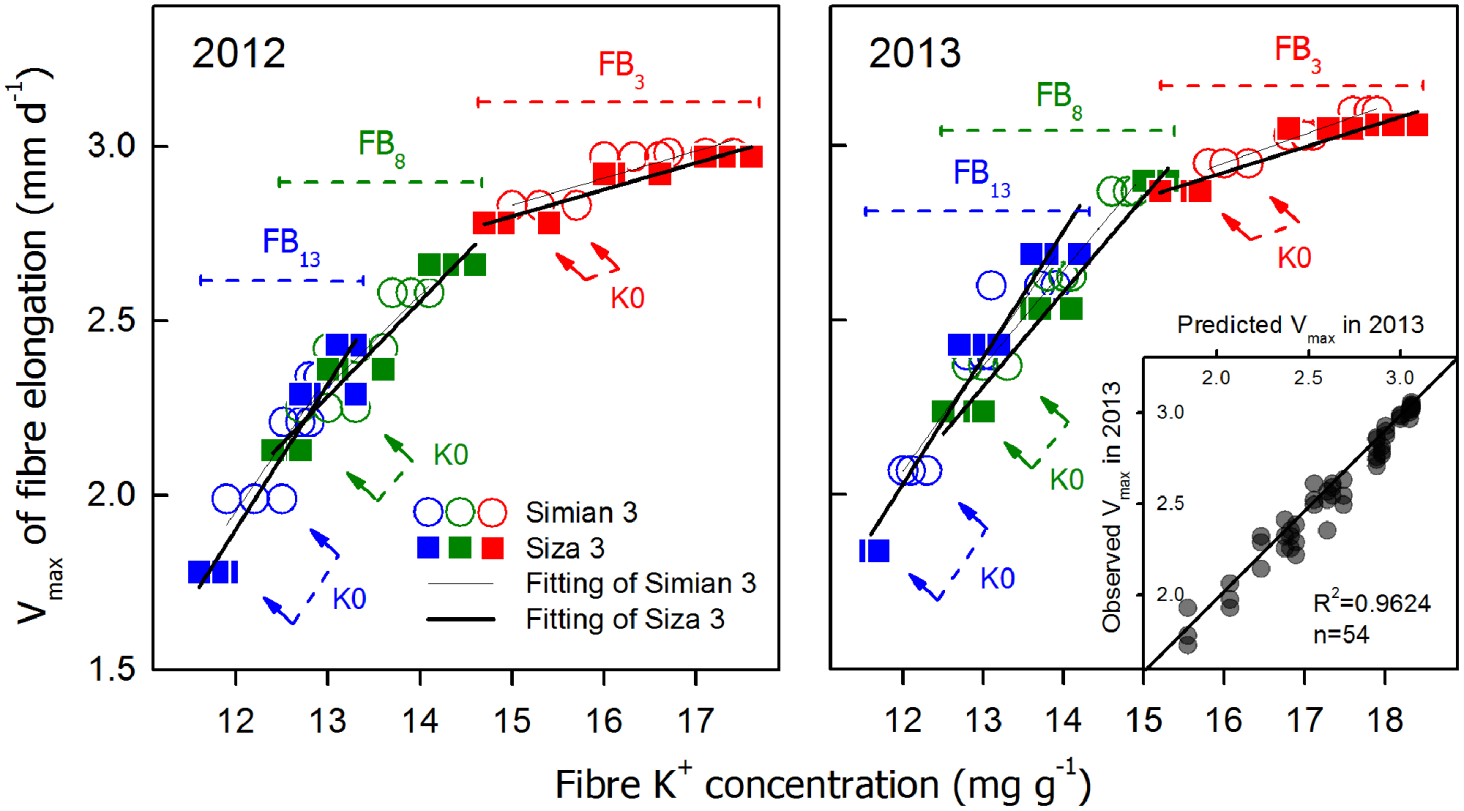
**Correlations between “*V*_max_ and fiber K^+^ concentration [[K^+^]_fiber_] at 10 DPA and the repeatability between the two test years (insert).** The linked arrows indicate the large difference between the two cultivars in their K^+^ accumulation ability under K0.

**FIGURE 9:**
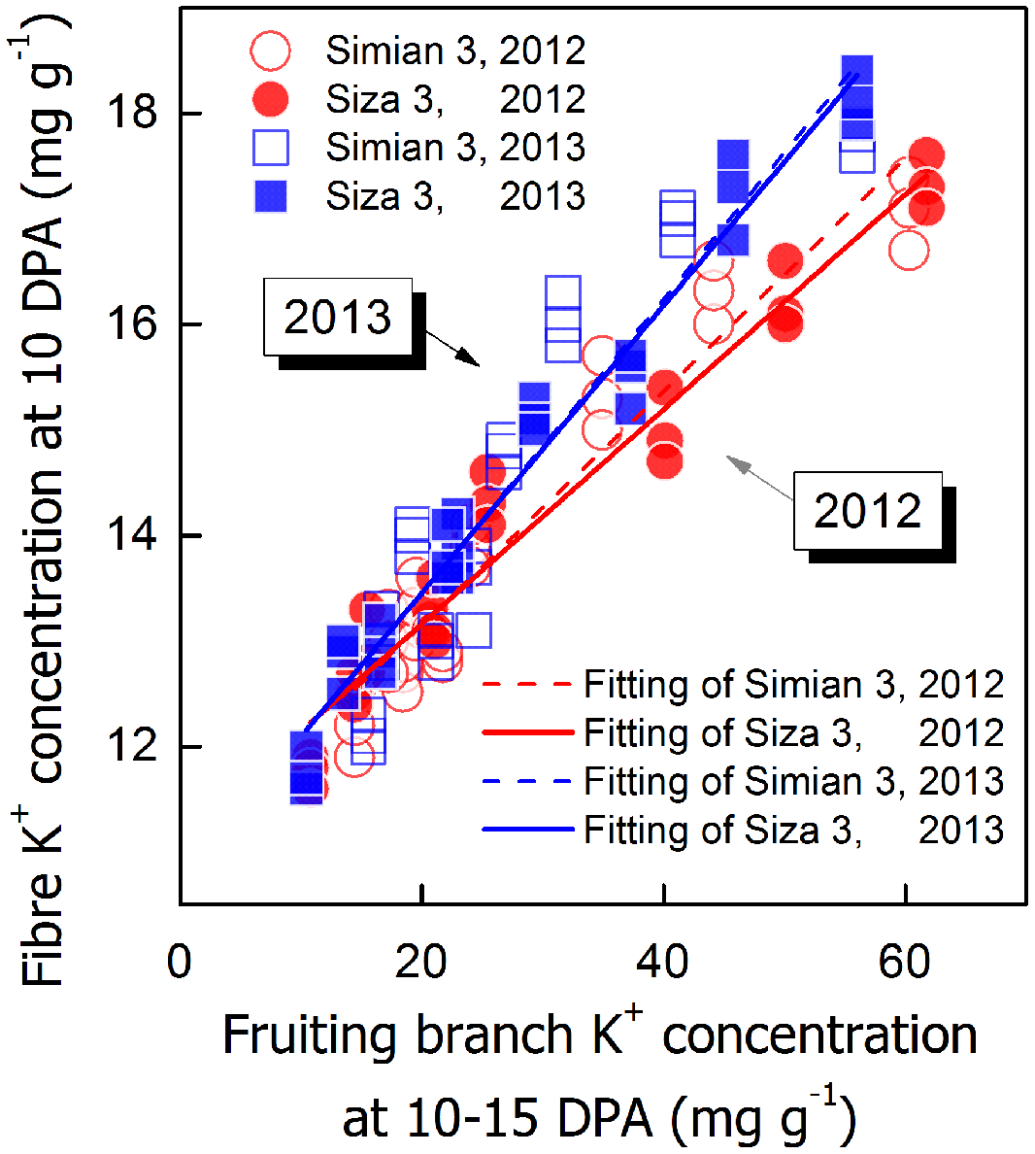
**Correlations between fiber K^+^ concentration ([K^+^]_fiber_) at 10 DPA and FB K^+^ concentration ([K^+^]_FB_) at 10–15 DPA**.

**FIGURE 10:**
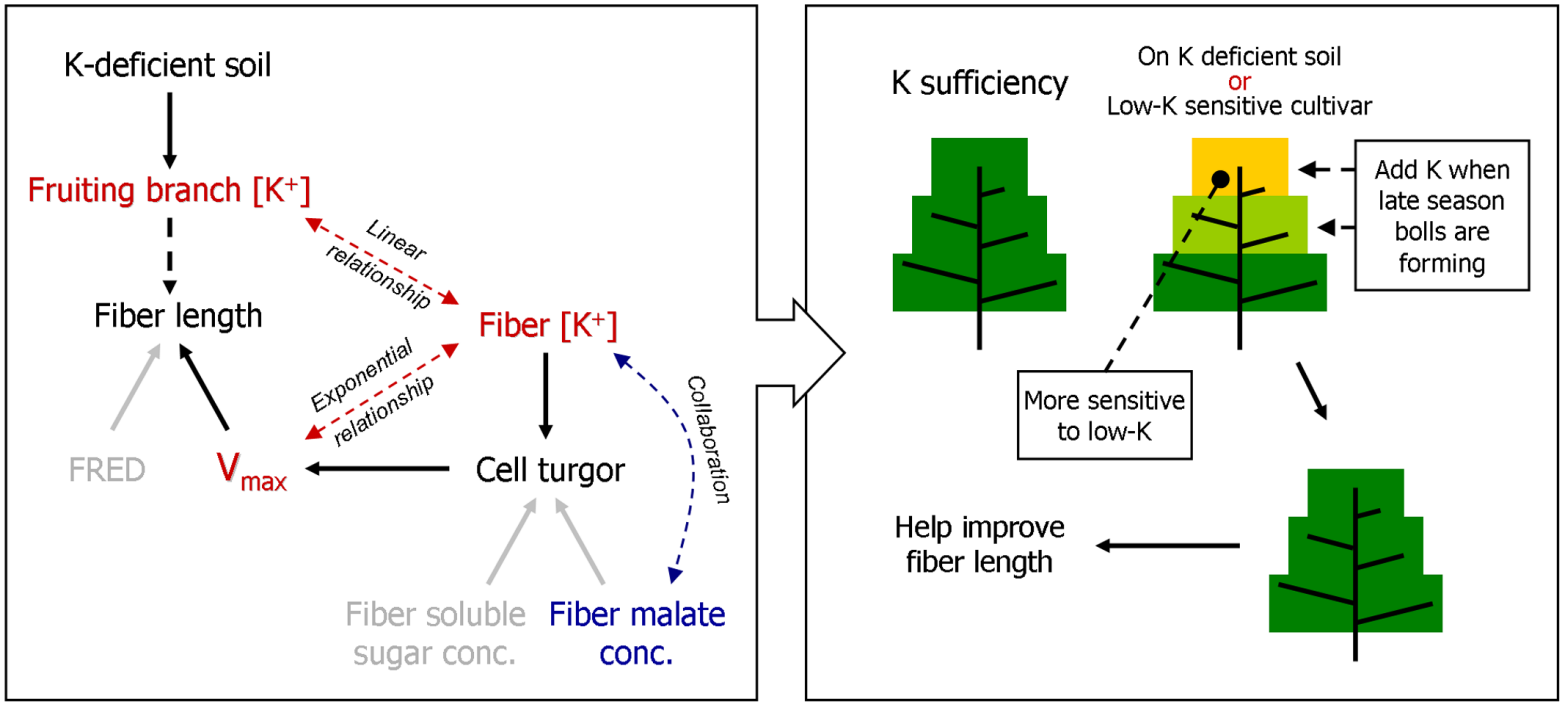
**A model of how FB K^+^ concentration [[K^+^]_FB_] regulates fiber elongation by osmoregulation, and a proposed K fertilization scheme based on it.** FRED, fiber rapid elongation duration; *V*_max_, the maximum velocity of fiber elongation.

The dynamic change in fiber length was regressed by the following logistic function (1) (Naithani et al., 1982; Zhao et al., 2012), in which *Len* represents fiber length. *Len_m_* is the theoretical maximal fibre length, which is a parameter in this model along with *a* and *b*.

(1) $$Len=\frac{{Len}_{m}}{1+a\times e^{b\times DPA}}$$

Eigenvalues, *V*_max_ (the maximum velocity of fiber elongation) and *FRED* (fiber rapid elongation duration), were derived from formulae (2) and (3) (Zhao et al., 2012), in which *DPA*_REI_ represents the initiation DPA of rapid elongation, and *DPA*_RET_ represents the termination DPA.

(2) $$V_{\max}=-\frac{b\times {Len}_{m}}{4}$$

(3) $$\begin{array}{cc} FRED & ={DPA}_{REI}-{DPA}_{RET}\\ & =\frac{1}{b}\times In\frac{2-\sqrt{3}}{a}-\frac{1}{b}\times In\frac{2+\sqrt{3}}{a}\\ & =\frac{1}{b}\times In\frac{2-\sqrt{3}}{2+\sqrt{3}}\\ & \approx -2.6339\times \frac{1}{b} \end{array}$$

*CV* = Standard deviation (*SD*)*/Mean* × 100%.

*CS* = [*Len*_fibre(K2500)_*-Len*_fibre(K0)_]*/Len*_fibre(K250)_ × 100%, in which *“Len*_fibre_*”* represents fiber length. The formula can also be written as [*C*_i(K250)_*-C*_i(K0)_]*/C*_i(K250)_ × 100%, in which *“C*_i_*”* represents the concentration of fiber K^+^ or malate or soluble sugar at 10 DPA.

## Results

### K^+^ Levels in Soil, Cotton Fruiting Branches, and Fiber Cells

Soil K deficiency induced symptoms in leaves that corresponded with previous studies (Oosterhuis, 1995; Wright, 1999), indicating that our field experiment met the expectation that K deficiency was severe enough to affect the growth of cotton lint (Dong et al., 2004). K-deficient leaves do not produce enough carbohydrate through photosynthesis for the heavy boll load of cotton (Wright, 1999); visible differences in final fiber length were observed among the K treatments (**Figure 1B**).

In 2013, there were 43 days with an MDT (mean daily temperature) over 30°C, twice the 21 days in 2012 (**Figure 1C**). The total rainfall from 25th April to 1st November in 2013 was 720.8 mm, which exceeded the 601.2 mm in 2012. Torrential rain occurred on 9th Aug 2012 with a total rainfall of 126.3 mm, and the relatively higher MDT combined with the more regular rainfall in 2013 improved cotton growth over 2012. This was reflected in a slower reduction in [K^+^]_FB_ (FB K^+^ concentration) in the control plants (K250 treatment, **Figure 2A**).

The [K^+^]_FB_ continued decreasing from the flowering to the boll-opening stage, and the three fruiting branches possessed distinct [K^+^]_FB_ curves (**Figure 2A**). Under K deficiency, significantly lower [K^+^]_FB_ were found in the low-K-sensitive cultivar, *Siza 3*, on the 13th FB (see the red and green lines in **Figure 2A**). Arrows on the [K^+^]_FB_ curve indicate a date within 10–15 DPA of the corresponding bolls (**Figure 2A**), when fibers reached peak velocity of elongation (**Figure 4**). In other words, it was the period when [K^+^]_FB_ most likely affected fiber elongation through osmoregulation (**Figure 6A**).

Fiber length varied from one FB to another, but bolls on the upper fruiting branches had longer fibers (**Figure 3A**). Meanwhile, the fiber lengths of the bolls on the upper fruiting branches decreased more when under severe K deficiency (**Figure 3A**). Generally speaking, *cv. Siza 3* fibers were longer than those of *cv. Simian 3* under the K-sufficient condition, but they suffered a greater reduction in length when K supply became insufficient, especially on the upper fruiting branches (**Figure 3A**). By analyzing the correlation between final fiber length and [K^+^]_fiber_ (10 DPA) or [K^+^]_FB_ (10–15 DPA), both [K^+^]_fiber_ and [K^+^]_FB_ were found to have strong positive effects on final fiber length on all of the sampled fruiting branches (**Figures 3B,C**). Most of the relationships were linear except those between fiber length and the [K^+^]_FB_ of the third FB for *cv. Simian 3* in 2012 and 2013. However, inconspicuous relationships among fruiting branches could have been present, so further analysis of fiber elongation was needed to determine why [K^+^]_fiber_ and [K^+^]_FB_ regulated fiber length so efficiently.

### Characteristics of Fiber Elongation Process

The elongation process reached maximum velocity at approximately 10–15 DPA, and fiber length reached a peak value after 24 DPA. These data fit a sigmoidal model very well (**Figure 4**), and the eigenvalues *V*_max_ (maximum velocity of elongation), FRED (fiber rapid elongation duration), and *Len*_max_ (maximum final fiber length) were calculated from the fitted logistic function (**Table 1**). In the current study, in which fiber length was directly influenced by the varying [K^+^]_FB_, it seems that both *V*_max_ and FRED have been modified (**Table 1**). Through the CV analysis, we found that *V*_max_ rather than FRED was readily influenced by [K^+^]_FB_ (left in **Figure 5**). From the analysis of the impact of FB node on these two eigenvalues (right in **Figure 5**), FRED was preferentially influenced by the “FB / Temp.” factor instead of [K^+^]_FB_. We used “FB/Temp.” to represent the change in FB node and the correspondingly different boll period temperatures. In other words, the variation in fiber length from bolls from different seasons was mainly caused by their different positions on the plant and boll period temperature. Therefore, *V*_max_ was selected as a dominant eigenvalue regulating the fiber elongation process under low-[K^+^]_FB_ stress, and focusing on *V*_max_ rather than FRED in the following analysis helped us remove the interference from the FB.

**Table 1:** Main eigenvalues (*V*_max_, FRED, *Len*_max_) of the fiber elongation process logistic function.

Cultivar	FBN	K treatment	*n*	2012					2013				
				*R*^2^	*V*_max_ (mm d^-1^)	FRED (d)	*Len*_max_ (mm)	*Len*_obs_ (mm)	*R*^2^	*V*_max_ (mm d^-1^)	FRED (d)	*Len*_max_ (mm)	*Len*_obs_ (mm)
*Simian 3*	13th	K0	8	0.9929	2.0	8.8	26.7	27.0 c	0.9969	2.1	8.6	26.9	27.4 c
		K125	8	0.9973	2.2	8.4	28.3	28.6 b	0.9980	2.4	7.8	28.5	28.6 b
		K250	8	0.9976	2.3	8.3	29.5	30.1 a	0.9994	2.6	7.6	29.9	30.1 a
	8th	K0	9	0.9987	2.2	7.6	25.8	25.9 c	0.9990	2.4	7.2	25.8	25.8 b
		K125	9	0.9988	2.4	7.4	27.1	27.2 b	0.9998	2.6	6.7	26.8	27.0 ab
		K250	9	0.9994	2.6	7.1	27.9	28.3 a	0.9999	2.9	6.5	28.1	28.3 a
	3rd	K0	7	1.0000	2.8	5.8	24.9	25.0 b	0.9992	2.9	5.8	25.8	25.3 b
		K125	7	0.9999	3.0	5.8	26.0	26.0 ab	0.9997	3.0	5.8	26.7	26.4 a
		K250	7	0.9999	3.0	5.8	26.4	26.6 a	0.9999	3.1	6.0	27.9	27.0 a
*Siza 3*	13th	K0	8	0.9998	1.8	10.0	27.1	27.6 c	0.9979	1.8	9.5	26.6	27.2 c
		K125	8	0.9990	2.3	8.6	29.9	30.2 b	0.9981	2.4	7.8	28.8	29.2 b
		K250	8	0.9980	2.4	8.5	31.3	31.6 a	0.9979	2.7	7.5	30.9	31.3 a
	8th	K0	9	0.9998	2.1	8.0	25.8	25.7 b	0.9998	2.2	7.6	25.9	26.2 c
		K125	9	0.9997	2.4	7.8	27.9	28.6 a	0.9994	2.5	7.3	28.2	28.6 b
		K250	9	0.9976	2.7	7.2	29.1	29.8 a	0.9995	2.9	6.8	29.8	30.5 a
	3rd	K0	7	0.9999	2.8	6.0	25.2	25.7 b	0.9997	2.9	5.8	25.3	25.6 b
		K125	7	0.9999	2.9	6.0	26.4	26.8 ab	0.9999	3.0	5.8	26.8	26.8 ab
		K250	7	0.9997	3.0	6.0	27.2	27.5 a	0.9993	3.1	5.9	27.6	27.7 a

*FBN, fruiting branch node; V_max_, the maximum velocity of fiber elongation; FRED, fiber rapid elongation duration; Len_max_, the theoretical maximal fiber length; Len_obs_, the observed final fiber length. Data followed by different letters (a, b, c) in one cultivar × FB group represent statistically significant differences at p < 0.05 level based on ANOVA.*

### Osmotically Active Solutes in Cotton Fiber

Cell turgor is achieved through the influx of water, which is driven by the accumulation of osmotically active solutes including K^+^, malate and soluble sugar (Ruan et al., 1997). By measuring the changes in their concentrations during rapid fiber elongation (5–24 DPA), we found that their peak values emerged at 10 DPA for K^+^ and malate and at 5–10 DPA for soluble sugar (**Figure 6A**). This coincided with the period of *V*_max_ (**Figure 4**). Moreover, [K^+^]_fiber_ exhibited greater variation than the other two solutes, and there was a greater change in [K^+^]_fiber_ in *Siza 3* when switching from K sufficiency to deficiency (**Figure 6B**). These results indicate that K deficiency mainly altered the K^+^ content in elongating fibers compared with other osmotic solutes, and this pattern was more obvious in the low-K sensitive genotype. More interestingly, the malate content in the fibers of *cv. Simian 3* was consistently higher than that in *cv. Siza 3* (**Figure 6B**) regardless of the FB or K treatment in which it occurred. This reflects a possible connection between [K^+^]_fiber_ and malate, and that malate might play a role in promoting [K^+^]_fiber_ accumulation in severely K-deficient soil in the low-K-tolerant cultivar, which could be meaningful for evaluating a genotype’s capacity for coping with K deficiency. Additionally, a quantitative comparison between concentrations of different osmotically active solutes using CS (CS; **Figure 7**) indicated that the effect of K deficiency (**Figure 2A**) on [K^+^]_fiber_ was 2.5–3.2 times (in 2012) and 2.0–2.6 times (in 2013) greater than on malate and 2.7–3.4 times (in 2012) and 2.1–2.8 times (in 2013) greater than on soluble sugar.

### Relationship Between K^+^ Level and Fiber Elongation

A series of correlations between *V*_max_ and [K^+^]_fiber_ (10 DPA) are shown in **Figure 8**. There were significant linear relationships between *V*_max_ and [K^+^]_fiber_ on each FB; interestingly, the regression lines of the two cultivars almost overlap (**Figure 8**). In contrast, these two cultivars also had very different *V*_max_ values under the K0 treatment, especially for the upper fruiting branches (see the linked arrows in **Figure 8**), and the different amounts of K^+^ available for fiber elongation between cultivars might be a crucial reason for this (**Figure 2A**). When all of the regression lines from the three fruiting branches were combined, an approximate exponential curve appeared (**Figure 8**), in which the upper fruiting branches tended to have lower [K^+^]_fiber_ while the lower fruiting branches tended to have higher [K^+^]_fiber_. Based on these results and a repeatability test between the two test years (insert in **Figure 8**), we found that *V*_max_ had an exponential relationship with the fiber peak K^+^ concentration when considering all three fruiting branches. This relationship could be successfully applied to both low-K-sensitive and low-K-tolerant upland cotton genotypes. Because this relationship varied little between the two test years, we combined the data from the 2 years and produced the following function (1), which is a relationship between *V*_max_ and fiber peak K^+^ concentration (for the current study, it was [K^+^]_fiber(10_ _DPA)_).

(4) $$\begin{array}{c} V_{\max}=3.17-146.48\times EXP\left(-0.4\times {\left[K^{+}\right]}_{fiber\left(10\,\;DPA\right)}\right)\\ R^{2}=0.9621^{**},\;n=108 \end{array}$$

To verify whether [K^+^]_FB_ could efficiently regulate [K^+^]_fiber_, a correlation analysis between [K^+^]_fiber_ and [K^+^]_FB_ was performed (**Figure 9**). Although the intensity of the influence of [K^+^]_FB_ on [K^+^]_fiber_ changed from year to year, the difference between the two genotypes remained small (**Figure 9**). Due to this linear regulation, it seems very likely that [K^+^]_FB_ regulated [K^+^]_fiber_ through the K^+^-osmotic pathways.

## Discussion

### K^+^ Plays a Vital Role in Cotton Fiber Growth Through Osmoregulation

A minimum K^+^ level in cotton tissue is essential for plant growth (Leigh and Wyn Jones, 1984) as well as the development of cotton fiber (Dhindsa et al., 1975). Soluble sugar, malate, and K^+^ were considered the main active solutes in rapidly elongating fibers (Dhindsa et al., 1975; Ruan et al., 1997), so the K^+^ status of a cotton plant is likely to regulate fiber elongation through the osmoregulatory pathway (Cassman et al., 1990). Based on our experiment, we verified the role of K^+^ in the osmoregulation between the FB and elongating fibers (**Figure 7**). An exponential relationship was found between *V*_max_, and [K^+^]_fiber_ (**Figure 8**), and a linear relationship was found between [K^+^]_fiber_ and [K^+^]_FB_ (**Figure 9**).

*V*_max_ and FRED are important eigenvalues in the fiber elongation process due to their abilities to change fiber length (Zhao et al., 2012), but which one plays the leading role during this process is not clear. *V*_max_ depends on cell turgor (Campas and Mahadevan, 2009), which reflects the force driving fiber elongation (Ruan et al., 2001; Fricke and Peters, 2002); FRED tends to be controlled by both the timing of secondary cell wall synthesis (Ruan, 2007; Wang and Ruan, 2010) and the structural change in the PD between the fiber cell and the epidermal cell (Ruan et al., 2001). In our study, the main effect of plant K^+^ status on fiber elongation was through the regulation of *V*_max_ (**Figure 5**). For the purpose of building the physiological connection between fiber elongation and the K^+^ status of the plant, we focused our analysis on *V*_max_, and the results indicated that the *V*_max_ could be osmotically adjusted by the plant K^+^ status (**Figures 8** and **9**).

### Fibers of Late Season Bolls are More Sensitive to K Deficiency

Cotton (*G. hirsutum*) has indeterminate growth, so in China, it fruits continuously from July to October. According to the growth in body size and the increasing boll amount, cotton bolls grown at different positions along the stem suffer from different nutritional (Boquet and Moser, 2003) and environmental conditions (Davidonis et al., 2004; Zhao et al., 2012). Late season bolls are more prone to K deficiency, which is reflected in K deficiency symptoms (Pettigrew et al., 1996; Read et al., 2006). According to our findings, upper fruiting branches have a greater potential to grow longer fibers, but their fiber length decreases more dramatically under K deficiency (**Figure 3A**). This might be caused by their poor nutrient competitiveness for K^+^ in plant (**Figure 2A**), which promotes K deficiency disease in the upper fruiting branches.

### Genotypic Differences in Fiber Elongation in Response to K Deficiency

The efficiency of K utilization varies from species to species, and great genotypic variation in K utilization can exist in a single species (Wang et al., 2012). In our study, we used two cultivars with distinct capacities for low-K tolerance (Yang et al., 2014). Cultivar *Siza 3*, the low-K-sensitive genotype, indeed had a more favorable fiber length trait than *cv. Simian 3* under K sufficiency (**Figure 3**), but it exhibited a greater decline in fiber length when conditions turned to K deficiency (**Figure 3**). Therefore, regardless of the intrinsic differences, *Siza 3* was more sensitive to K deficiency in terms of fiber length.

A correlation analysis between *V*_max_ and [K^+^]_fiber_ of the two cultivars was also performed, and the results show that *cv. Siza 3* exhibited lower [K^+^]_fiber_ than *cv. Simian 3* under K-deficient conditions (**Figure 8**). This is the possible reason why *cv. Siza 3* had smaller *V*_max_ values compared with *cv. Simian 3*, especially under the K0 treatment. The genotypic difference in [K^+^]_fiber_ between two cultivars appears to be due to their differing capacities for absorbing K^+^ (**Figures 2** and **9**). Wang et al. (2012) reported similar results in terms of cotton biomass accumulation and photosynthesis activity; in their study, a K-efficient cultivar produced 66.7% more biomass than the K-inefficient cultivar when under K deficiency but similar biomass under K sufficiency.

Interestingly, low-K-tolerant *cv. Simian 3* tended to have a higher fiber malate content at 10 DPA compared with low-K-sensitive *cv. Siza 3*, and the pattern was constant across K treatments, fruiting branches, and years (**Figure 6B**). We suggest that this might explain the high capacity of *cv. Simian 3* for K^+^ accumulation (**Figure 2A**). Malate has a collaborative role with K^+^ in osmoregulation not only in the movement of guard cells (Allaway, 1973) but also cotton fiber elongation (Dhindsa et al., 1975), and the malate accumulating in guard cells can neutralize at least half of the potassium (Raschke and Schnabl, 1978). Hence, the phenomenon of higher malate content in fibers suggests a mechanism through which the low-K-tolerant genotype has stronger capacities to accumulate K^+^.

### Suggestions for Cotton Management Under K-Deficiency

K deficiency in soil (**Figure 2B**) negatively impacts K^+^ accumulation in cotton plants. The significantly declining [K^+^] in the fruiting branches affected the cotton bolls by presenting severely K-deficient growth conditions (**Figure 2A**), and this decline in [K^+^]_FB_ led to a reduction in fiber length (**Figures 3A** and **4**) by altering the *V*_max_ during the fiber elongation process (**Figure 10**). According to our study, [K^+^]_fiber_ was the factor that affected *V*_max_ (**Figure 8**) and was regulated by [K^+^]_FB_ (**Figure 9**) through K^+^-osmoregulation. This indicates that elevating [K^+^]_FB_ above a critical value may help to grow longer fibers. However, upper fruiting branches are more prone to K deficiency disease due, basically, to a larger reduction in [K^+^]_FB_ compared with lower fruiting branches (**Figure 2A**). Therefore, adding extra potash fertilizer or allocating it for the development of late season bolls might enhance the K^+^ level of the upper fruiting branches and result in grow longer fibers. This K fertilization scheme applies to cotton cultivation on K-deficient soil and to the growth of low-K-sensitive cotton cultivars.

## Author Contributions

JY collected and analyzed data, and wrote and revised this manuscript; JY and WH completed the cotton plant nutrition determination; WH, WZ, BC, YW, YM, and ZZ helped analyze data and assisted in revising the manuscript; YM and ZZ designed the experiments together and final approved to publish this version. All authors have participated in this study and approved to publish this manuscript.

## Conflict of Interest Statement

The authors declare that the research was conducted in the absence of any commercial or financial relationships that could be construed as a potential conflict of interest.

## Abbreviations

CS: coefficient of stress
CV: coefficient of variation
DPA: days post anthesis
FB: fruiting branch
FRED: fibre rapid elongation duration
[K^+^]_FB_: fruiting branch K^+^ concentration
[K^+^]_fibre_: fibre K^+^ concentration
[K^+^]_soil_: soil soluble K^+^ concentration
*Len*_max_: the theoretically maximal fibre length
*Len*_obs_: the observed final fibre length
Temp.: temperature
*V*_max_: the maximum velocity of fibre elongation
