# Supplementary figures and images for: Fruiting Branch K+ Level Affects Cotton Fiber Elongation Through Osmoregulation
Source: Front Plant Sci. 2016 Jan 22;7:13. doi: 10.3389/fpls.2016.00013 (PMC4722289; doi:10.3389/fpls.2016.00013)

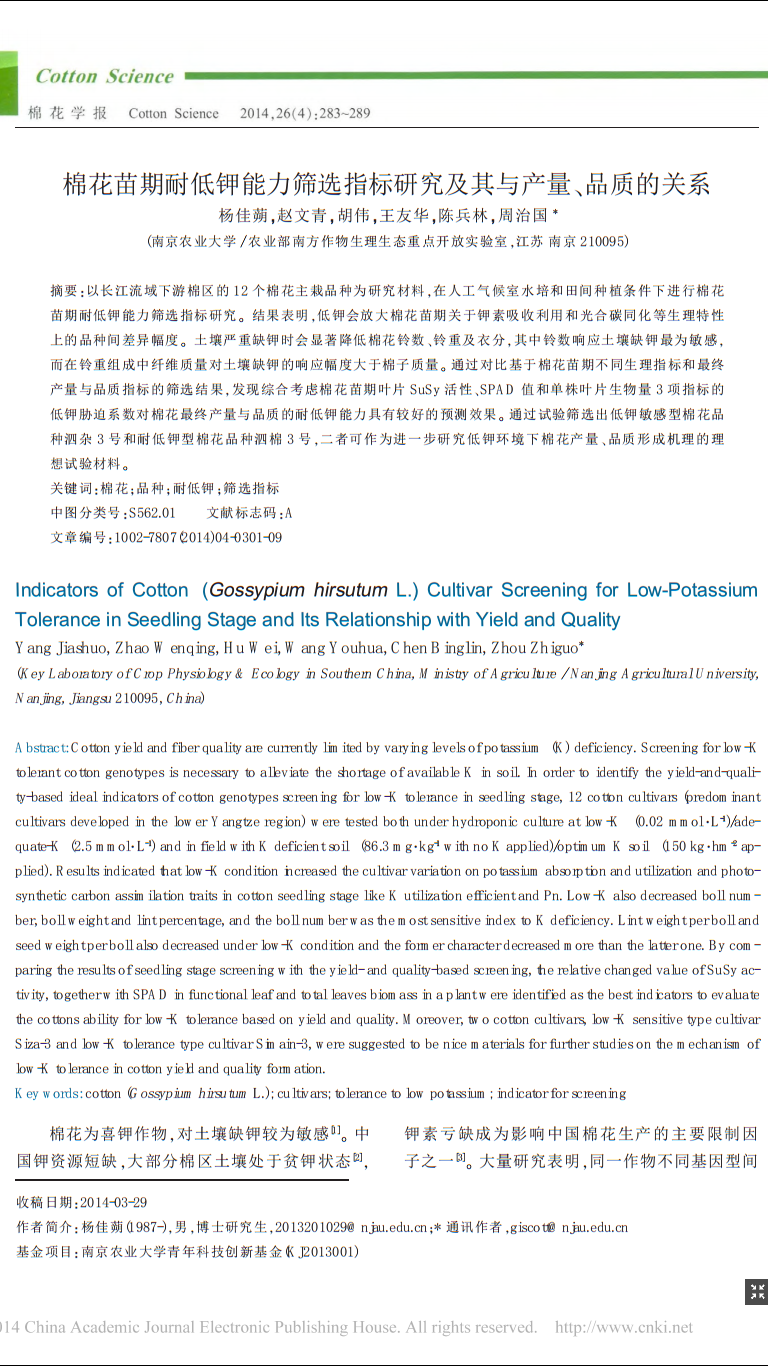

Supplement: Supplementary file 1 [file Image_1.TIF]
